# Cognitive impairment assessed by Mini-Mental State Examination predicts all-cause and CVD mortality in Chinese older adults: A 10-year follow-up study

**DOI:** 10.3389/fpubh.2022.908120

**Published:** 2022-11-28

**Authors:** Zhiqiang Li, Xinran Gong, Shengshu Wang, Miao Liu, Shaohua Liu, Yanding Wang, Di Wu, Meitao Yang, Rongrong Li, Haowei Li, Xuehang Li, Shimin Chen, Xiushan Zhang, Ruizhong Jia, Jinpeng Guo, Yao He, Yong Wang

**Affiliations:** ^1^School of Public Health, China Medical University, Shenyang, China; ^2^Chinese People's Liberation Army Center for Disease Control and Prevention, Beijing, China; ^3^Beijing Key Laboratory of Aging and Geriatrics, National Clinical Research Center for Geriatrics Diseases, Institute of Geriatrics, Second Medical Center of Chinese People's Liberation Army General Hospital, Beijing, China; ^4^Department of Healthcare, Agency for Offices Administration, Central Military Commission, Beijing, China; ^5^Department of Epidemiology and Statistics, Graduate School of Chinese PLA General Hospital, Beijing, China; ^6^State Key Laboratory of Kidney Diseases, Department of Epidemiology, Chinese People's Liberation Army General Hospital, Beijing, China

**Keywords:** cognitive impairment, older adults, MMSE, mortality, follow-up

## Abstract

**Objective:**

Cognitive impairment (CI) has been demonstrated as a useful proxy measure of mortality in Western populations. However, the predictive value of CI in Chinese populations is unknown. We aimed to explore whether CI is independently associated with increased long-term all-cause and cardiovascular disease (CVD) mortality in Chinese older adults and the association of performance in specific MMSE sub-domains to subsequent mortality.

**Methods and results:**

A total of 4,499 older adults [mean (SD) age, 70.3(6.7) years] who received a sample investigation from 2011 to 2014 were followed up till 2021 for mortality. The Mini-Mental State Examination was used to assess cognitive function, and Cox's proportional hazard models were used to evaluate the effects of cognitive function on the risk of all-cause and CVD mortality. Demographic characteristics, lifestyle, and health status were included as covariates. During a 10-year follow-up, a total of 667 (14.8%) died. In the fully adjusted model, compared with cognitively normal participants with CI had a 1.33-fold [HR, 1.33; (95% CI, 1.10–1.61)] greater risk of all-cause mortality and a 1.45-fold [HR, 1.45; (95% CIs, 1.11–1.92)] greater risk of CVD mortality. After a similar multivariable adjustment, a per-SD increase in MMSE scores was associated with a reduced risk of all-cause mortality [HR, 0.85; (95% CI, 0.78–0.93)] and CVD mortality [HR, 0.74; (95% CI, 0.65–0.84)]. In the unadjusted model, MMSE sub-domains (apart from immediate recall) were associated with mortality. But only orientation and calculation and attention were still independently associated with all-cause and CVD mortality in a multivariable model.

**Conclusion:**

These findings confirmed that CI is a marker of all-cause and CVD mortality risk in Chinese older adults, independently of other commonly assessed risk factors, and some sub-domains of the MMSE may have stronger associations with mortality. Further research is needed to identify the mechanisms underlying the observed associations.

## Introduction

The aging of the population is a significant public health concern worldwide, especially in China. According to the statistical yearbook issued by the Chinese government (1), older adults (≥65 years) accounted for 13.5 % of the total population in 2020. Life expectancy continues to increase, and the average life expectancy is expected to be higher than 80 years in 2030. However, dementia, marked by significant cognitive impairment (CI), reduces the lifespan expectancy and quality of life of older adults (2). CI has increased in the past decades, and the latest national prevalence estimates for 2015–2018 were 15.5%, representing about 38.77 million patients in China (3). The number of deaths attributed to Alzheimer's disease (AD) and other types of dementia in China has increased from 1.6 million in 1990 to 2.3 million in 2017 (4).

Cognitive impairment is a highly prevalent mental disorder and is considered a transitional stage between unimpaired cognition and dementia in older adults (3). It is a predictor of all-cause and CVD mortality, with the majority of studies having been conducted in high-income countries (especially for cause-specific mortality) (5–10). Some studies in Germany and Finland found an association between cognitive function and CVD mortality when adjusting for age and sex, but not when additionally adjusting for cardiovascular conditions and biomarkers in multivariable models (11–13). CVD is the leading cause of death in China, being the cause of 36.0% of deaths in the Chinese population (14). Given the high disease burdens and the huge socioeconomic burden caused by CVD (14), clarifying the relationship between CI and CVD mortality risk is important. Most previous studies in China examined the relationship between CI and all-cause mortality, and several studies had the limitations of including a narrow age range of older adults (aged 80 or above) (13, 15, 16), which may be more prone to a “survivor bias” (17, 18). Some studies did include a broader age range of older adults (19, 20), but these studies only focused on the association between CI and all-cause mortality. Moreover, these studies were limited by short follow-up time (13, 16), high rates of loss to follow-up (13), inadequate adjustment of potential explanatory factors (13, 15, 16), or failure to use education-adjusted MMSE cutoff scores (15, 20), which may limit the generalizability of the findings.

Epidemiological studies have reported the association between CI and mortality risk, but the link between MMSE sub-domains and mortality in different countries and regions was inconsistent (21–24). These studies have found an association between some sub-domains and mortality, but these sub-domains were not identical (21, 23, 24). Furthermore, the relationship between these domains and CVD mortality risk was less known, particularly in Chinese older adults. Just one known previous study in urban community-dwelling Chinese older adults reported an association between MMSE sub-domains and all-cause mortality risk, a finding that MMSE sub-domains (orientation to time, attention and calculation, recall, and language) were significantly associated with all-cause mortality only when studied in an unadjusted model, whereas there was no sub-domain that was associated with mortality when adjusting for gender and age (20). Therefore, using data from the Beijing Elderly Comprehensive Health Cohort study (BECHCS), we aimed to further examine the relationship between CI and the risk of all-cause and CVD mortality in Chinese older adults, taking into account a comprehensive range of covariates, and examining associations between MMSE sub-domains and mortality risk.

## Methods

### Study design and population

The Beijing Elderly Comprehensive Health Cohort study is a prospective cohort study, which is described in detail elsewhere (25, 26). Briefly, this representative cohort was based on a two-stage stratified random-clustering sampling method. Two districts representing the urban and rural areas of Beijing were selected to constitute the sample. All participants of the study sample accounted for about 10% of the total elderly, including the geographic distribution, age, gender, and education of the study sample, provided by the Ministry of Civil Affairs.

A total of 4,499 participants who had completed cognitive assessments were included in the baseline survey. The process of inclusion and exclusion of participants and how the follow-up procedure was performed is shown in Supplementary Figure S1. All eligible participants were informed about the details of the research and signed informed consent before the investigation. The Ethics Committee of the Chinese PLA General Hospital approved the study protocol (Ethics Number: S2021-327-01).

### Data collection

All doctors and nurses involved in the fieldwork received comprehensive training. Baseline data were collected by using standardized questionnaires and a face-to-face interview. Investigation contents included demographics, lifestyle, family history, and chronic conditions. The participants accepted a physical examination, including height, weight, waist circumference, and blood pressure. Blood pressure was measured two times using an aneroid sphygmomanometer with the participant after a 30-min rest in the seated position. Fasting blood samples of all participants were used for analyses. The specimens collected in urban regions were sent to the central certified laboratory of the Chinese PLA General Hospital in < 30 min, while samples in rural regions were transported within 90 min. Detailed information on data collection is found in Supplementary Table S2.

### Cognitive function assessment

The Mini-Mental Status Examination (MMSE) is a sensitive and effective screening tool, which has been used widely to evaluate cognitive function (19, 26). The MMSE scores range from 0 to 30, and it is clustered into six domains measuring different cognitive processes: time orientation (5 scores), place orientation (5 scores), calculation and attention (6 scores), immediate recall (3 scores), delayed verbal recall (3 scores), and language and others (8 scores). The lower the MMSE scores, the worse the cognitive function. This study used the Chinese version of MMSE, which has been validated in the Chinese population (19). Education-adjusted MMSE cut point scores were used to define CI based on relevant literature (13, 15): MMSE < 18 for uneducated participants, MMSE < 20 for participants with primary education level, and MMSE < 25 for participants with secondary school education level or above.

### Outcome event

The data on the survival status and the cause-specific death were ascertained from the Chinese Center for Disease Control and Prevention, which were verified by the medical insurance system or the public security department. The survival time of participants was measured from the date of the baseline interview from 2011 to the date of death or the date of the last follow-up (March 2021).

To protect the anonymity of participants, the causes of death were determined according to the tenth International Classification of Disease (ICD-10). These classifications included CVD (codes I00–I99) and “other causes.” In this study, the primary outcomes were all-cause mortality and CVD mortality.

### Covariates

Other co-variables collected during the baseline interview included demographic factors [age, gender, residence, educational level, and marital status], lifestyle information [smoking status, alcohol consumption, and exercise], multimorbidity, body measurement indicators [body mass index (BMI), waist circumference(WC), systolic blood pressure (SBP), and diastolic blood pressure (DBP)], and biochemical indexes [fasting plasma glucose (FPG), triglyceride (TG), total cholesterol (TC), glycosylated hemoglobin A1c (HbA1c), high-density lipoprotein cholesterol (HDL-C), low-density lipoprotein cholesterol (LDL-C), and uric acid].

Detailed measurement and classification of each variable were reported (25, 26). Current smoking status was defined as someone who currently smoked at least one cigarette per day; current alcohol drinking was defined as drinking alcohol at least once a week in the past year. BMI was calculated as weight (kg) divided by the square of height in meters (kg/m^2^). According to six activities of daily living (such as dressing, bathing, and eating), we divided physical function into either a complete functioning group or a physical impairment group. Hypertension was defined as SBP ≥140 mm Hg or DBP ≥90 mm Hg, or using antihypertensive drugs regularly. Diabetes was defined as those with FBG level ≥7.0 mmol/L, or the regular intake of hypoglycemic medication. Dyslipidemia was diagnosed as a serum total cholesterol level of ≥6.22 mmol/L, or LDL-C of ≥3.36 mmol/L, or HDL-C of ≤ 1.04 mmol/L, or drugs regulating dyslipidemia were used regularly. All chronic diseases (including COPD, hypertension, diabetes, coronary heart disease, dyslipidemia, stroke, and tumor) were identified according to self-reporters, and multimorbidity was defined as participants who had two or more chronic diseases at the same time.

### Statistical analysis

Data were presented as frequency (percentage) for categorical variables and as means (SD, standard deviation) or medians (IQR, interquartile range) for continuous variables. The differences between groups were tested by ANOVA or Mann–Whitney U-test for continuous variables and the chi-square test for categorical variables. SPSS for Windows (20.0, Chicago, IL) and R version 3.6.3 were used to analyze the data. All statistical tests were two-tailed, and *p*-values of < 0.05 were considered statistically significant.

**Figure 1:**
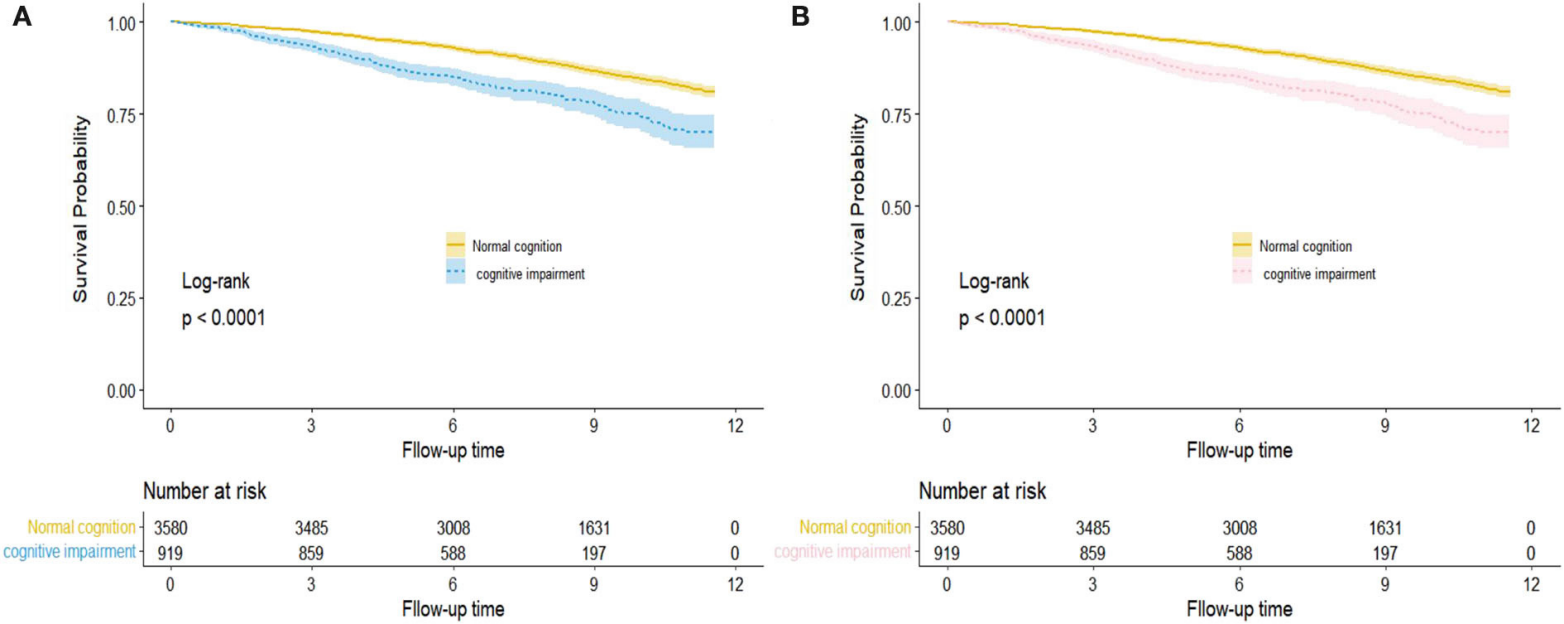
Kaplan–Meier survival curve for hazard of all-cause **(A)** and CVD **(B)** mortality by cognition function status. The areas of shadow represent 95% CIs.

The Kaplan–Meier survival curve revealed outcome trajectories in different groups for all-cause and CVD mortality (log-rank *p* < 0.001, Figure 1). The median follow-up time was estimated by using the reverse Kaplan–Meier survival curve, which was used to describe the follow-up in the censored population. The association between cognitive function and risk of all-cause and CVD mortality was estimated using Cox proportional hazard models. Hazard ratio (HR) and 95% confidence intervals (CIs) were reported. A series of models were established to adjust covariates and estimated the risk of all-cause and CVD mortality associated with the MMSE score per 1-SD increase change in the following three Cox proportional hazard models. Model 1 was the crude model; age (continuous), gender (men vs women), and residence (urban vs rural) were adjusted in model 2; education level (0/1-6/>6), marital status (married, widowed, and others), smoking status (current/former/never), alcohol drinking (current/former/never), and exercise (< 1 or ≥1 h/d) were added in model 3 based on model 2; physical impairment (yes/no), dementia history (yes/no), multimorbidity (yes/ no), BMI, WC, SBP, DBP, TG, HDL-C, LDL-C, FPG, HbA1c, and uric acid (all continuous) were further adjusted in model 4. The risk analysis time of participants is the outcome variable, the number of years measured from the baseline interview to death or censoring. The MMSE scores and the scores of MMSE sub-domains were standardized to mean 0 and standard deviation [SD] 1, to estimate the risk of all-cause and CVD mortality.

According to the previous reasearch (10, 13, 27), age and gender play a role in the relationship between CI and mortality. To explore the impact of the interaction between CI and more risk factors on mortality, we chose to perform stratified subgroup analysis by age, gender, residence, hypertension or not, and diabetes or not based on the fully adjusted Cox model above. The interactions between cognitive function and subgroup variables were tested to evaluate whether the combined effect of different subgroups was similar.

To further confirm the stability of our primary findings and assess potential reverse causality, we performed two sensitivity analyses: (1) Excluded participants died within 2 years of follow-up. (2) The association between cognitive function and the risk of all-cause and CVD mortality in participants with prior cardiovascular disease was further analyzed.

## Results

### Baseline characteristics

A total of 4,499 participants [mean (SD) age, 70.28 (6.73) years; 1815 (40.3%) men] who completed the initial MMSE were recruited in the baseline survey. The prevalence of CI was 20.43%, and the characteristics of the participants are shown in Table 1.

**Table 1:** Characteristics of participants according to different cognitive function status.

**Item**	**Normal cognition (*n* = 3,580)**	**Cognitive impairment (*n* = 919)**	***P* value**	**Total (*n* = 4,499)**
Age, years (mean; SD)	69.68 ± 6.18	72.68 ± 7.60	< 0.001	70.28 ± 6.73
Female, *n* (%)	2,067 (57.7)	617 (67.1)	< 0.001	2684 (59.7)
Residence, *n* (%)			< 0.001	
Rural	1,724 (48.2)	673 (73.2)		2,397 (53.3)
Urban	1,856 (51.8)	246 (26.8)		2,102 (46.7)
Marriage, *n* (%)			< 0.001	
Married	1,585 (44.3)	189 (20.6)		1,774 (34.9)
Widowed	1,696 (47.4)	657 (71.5)		2,353 (52.3)
Others	299 (8.4)	73 (7.9)		372 (8.3)
Education, years *n* (%)			< 0.001	
Illiteracy (0)	724 (20.2)	475 (51.7)		1,199 (26.7)
Primary school (1–6) Above primary school (>6)	120 (33.6) 1,653 (46.2)	220 (23.9) 224 (24.4)		1,423 (31.6) 1,877 (41.7)
Exercise, *n* (%)			< 0.001	
≥1 h/d	2,494 (69.7)	501 (54.5)		2,995 (66.6)
< 1 h/d	1,086 (30.3)	418 (45.5)		1504 (33.4)
Smoking status, *n* (%)			0.006	
Current smoker	628 (17.5)	150 (16.3)		778 (17.3)
Former smoker	499 (13.9)	95 (10.3)		594 (13.2)
Never smoker	2,453 (68.5)	674 (73.3)		3,127 (69.5)
Alcohol consumption, *n* (%)			0.785	
Current drinker	1,158 (32.3)	300 (32.6)		1,458 (32.4)
Former drinker	195 (5.4)	55 (6.0)		250 (5.6)
Never drinker	2,227 (62.2)	564 (61.4)		2,791 (62.0)
Physical impairment, *n* (%)	9 (0.3)	11 (0.2)	< 0.001	20 (0.4)
Dementia history, *n* (%)	162 (4.5)	22 (2.4)	0.004	184 (4.1)
Multimorbidity, *n* (%)	2,720 (76.0)	671 (73.0)	0.043	3,391 (75.4)
BMI groups, *n* (%)			0.010	
Underweight	103 (2.9)	44 (4.8)		147 (3.3)
Normal weight	1,415 (39.5)	376 (40.9)		1,791 (39.8)
Overweight Obese	1531 (42.8) 531 (14.8)	357 (38.8) 142 (15.5)		1888 (42.0) 673 (15.0)
WC (cm)	88.63 ± 8.65	88.47 ± 8.25	0.061	88.59 ± 8.57
BMI (mean; SD), kg/m^2^	24.73 ± 3.26	24.56 ± 3.17	0.149	24.70 ± 3.24
SBP (mmHg) (mean; SD)	135.98 ± 18.68	138.29 ± 20.21	0.001	136.45 ± 19.02
DBP (mmHg) (mean; SD)	78.53 ± 10.87	79.36 ± 11.88	0.043	78.70 ± 11.08
HDL-C (mean; SD)	1.41 ± 0.43	1.44 ± 0.45	0.045	1.41 ± 0.44
LDL-C (mean; SD)	3.06 ± 0.88	2.94 ± 0.86	0.001	3.03 ± 0.88
TC (mean; SD)	4.98 ± 1.04	4.93 ± 1.05	0.155	4.97 ± 1.04
TG, median (IQR)	1.34(0.96,1.88)	1.26(0.91,1.78)	0.009	1.32(0.95,1.85)
FPG, median (IQR)	5.56(5.14,6.28)	5.52(5.00,6.20)	0.002	5.55(5.11,6.26)
HbA1c, median (IQR)	5.80(5.40,6.10)	5.80(5.50,6.10)	0.813	5.80(5.40,6.10)
Uric acid (mean; SD)	301.43 ± 84.27	288.83 ± 84.62	< 0.001	298.86 ± 84.49
Status of survival, *n* (%)			< 0.001	
Alive	3,097 (86.5)	735 (80.0)		3,832 (85.2)
All-cause death	483 (13.5)	184 (15.8)		667 (14.8)

Multimorbidity refers to two or more chronic diseases (hypertension, cardiovascular disease, cerebrovascular disease, diabetes, respiratory disease, stroke, cancer, and vision disease) that coexist in the same older person.

IQR interquartile range; SD, standard deviation; BMI, body mass index; WC, waist circumference; SBP, systolic blood pressure; DBP, diastolic blood pressure; HDL-C, high-density lipoprotein cholesterol; LDL-C, low-density lipoprotein cholesterol; TC, total cholesterol; TG, triglyceride; FPG, fasting plasma glucose; HbA1c, Hemoglobin A1c.

Increasing age was related to CI. Compared with cognitively normal participants, participants who had CI were older, more likely to be women, living in a rural area, widowed, illiterate, had exercised less, and had a higher level of HDL-C, but significantly lower LDL-C, TG, uric acid, and multimorbidity. Moreover, other general characteristics are also shown in Table 1. All indicators of participants with different cognitive functions showed significant differences (P < 0.05) in all aspects but alcohol consumption, BMI, WC, and TC.

### Mortality based on cognitive function status

**Figure 2:**
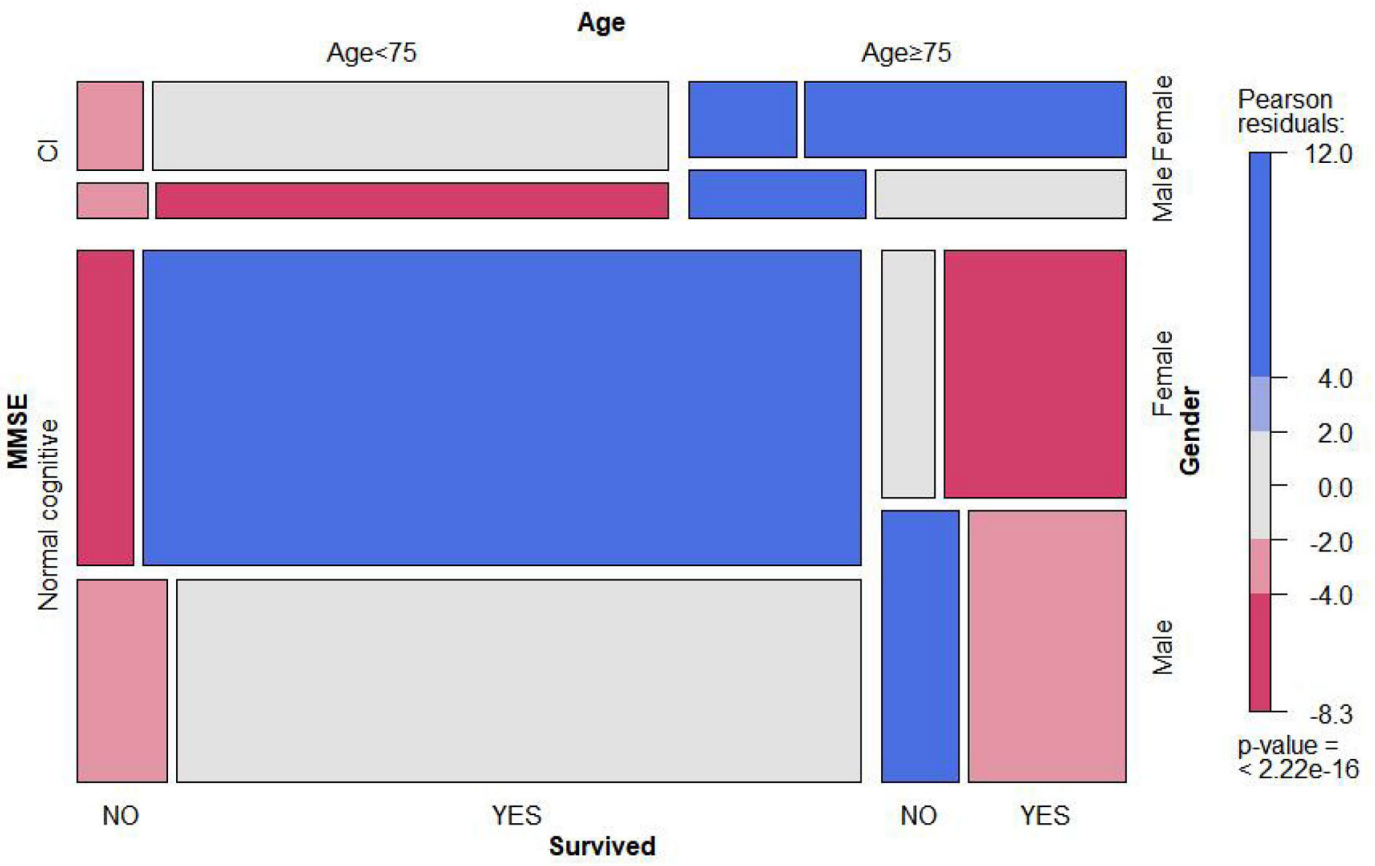
Mosaic matrix for MMSE, age, gender, and survival state. It is mainly used to visually display the observation frequency and proportion of combination of two or three classification variables in different dimensions. The specific value is seen as attachment Supplementary Table S3.

Out of the 4,449 participants collected at baseline, 667 (14.8%) participants died during a 10-year follow-up (Table 1) and 292 died due to CVD. Figure 2 shows the comparison of the observed proportion of four groups of classification variables, and the result displayed that the proportion of death in male groups and female groups over 75 years old increased significantly as the degree of cognitive function gradually decreased (*P* < 0.01). However, with the decline in cognitive function, the survival rate of female groups was significantly higher than that of male groups (*P* < 0.001). The specific data are attached to Supplementary Table S3.

### Baseline cognitive function and mortality

**Figure 3:**
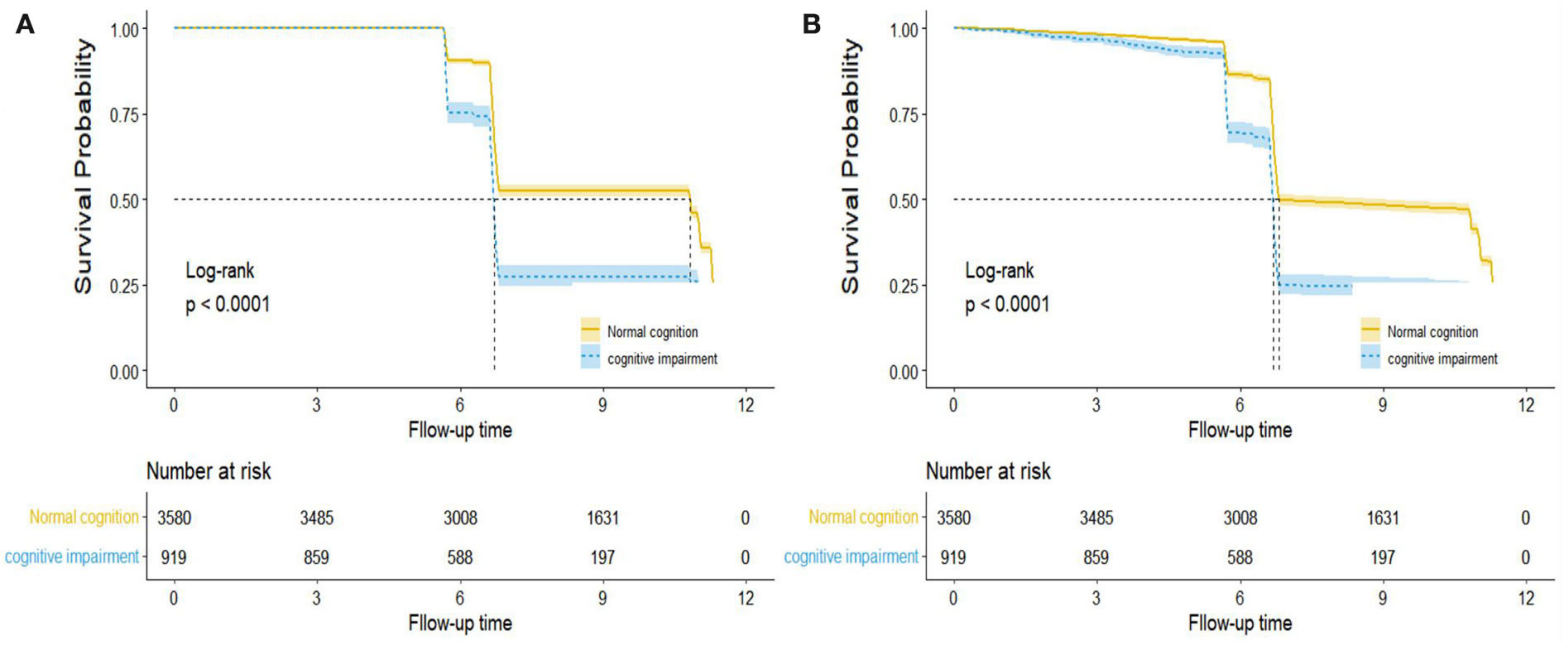
Reverse Kaplan–Meier survival curve for the median follow-up time by cognition function in all-cause **(A)** and CVD **(B)** mortality. Note: This is not the KM curve for the outcome events. In this plot, losses to follow-up were treated as “events” while the development of outcome events was treated as “censored.” The dashed line describes the median follow-up time, and the areas of shadow represent 95% CIs.

The reverse Kaplan–Meier survival curve estimated the median follow-up time of all-cause and CVD mortality based on cognition function status (log-rank *p* < 0.001, Figure 3). The median follow-up time for all-cause mortality was 10.83 years, and CVD mortality was 6.81 years in the normal cognition group. However, in the cognitive impairment group, the median follow-up for all-cause mortality vs. CVD mortality was 6.71 and 6.68 years, respectively.

The association between cognitive function and all-cause and CVD mortality risk is shown in Table 2. Compared with cognitively normal participants, CI was independently associated with all-cause and CVD mortality in both unadjusted and fully adjusted models. In the fully adjusted model, participants with CI had a higher risk of all-cause mortality (HR, 1.33; 95% CI, 1.10–1.61; *P* = 0.003). A similar finding was observed when we analyzed the association with CVD mortality (HR, 1.45; 95% CI, 1.11–1.92; *P* = 0.005). A per-SD increase in MMSE scores was associated with a 15% decreased risk of all-cause mortality and a 26% decreased risk of CVD mortality (Table 2).

**Table 2:** Hazard ratio for the association between cognitive function with all-cause and CVD mortality.

	**Model 1^a^**	**Model 2^b^**	**Model 3^c^**	**Model 4^d^**
	**HR (95%CIs)**	**HR (95%CIs)**	**HR (95%CIs)**	**HR (95%CIs)**
All-cause mortality				
Normal cognition Cognitive impairment	1(Reference) 1.99 (1.68–2.36) *	1(Reference) 1.37 (1.14–1.64)*	1(Reference) 1.32 (1.09–1.58) **	1(Reference) 1.33 (1.10–1.61)**
Per-SD increase	0.69 (0.65–0.74)*	0.80 (0.74–0.87)*	0.85 (0.78–0.93)*	0.85 (0.78–0.93)*
Cardiovascular disease mortality				
Normal cognition Cognitive impairment	1(Reference) 2.44 (1.90–3.13) *	1(Reference) 1.48 (1.13–1.94)**	1(Reference) 1.46 (1.11–1.92) **	1(Reference) 1.45 (1.11–1.92)**
Per-SD increase	0.60 (0.55–0.66)*	0.71 (0.63–0.79)*	0.73 (0.64–0.83)*	0.74 (0.65–0.84)*

CIs, confidence intervals; SD, standard deviation. CVD, cardiovascular disease. Hazard ratio (95% CI) was calculated from Cox models. When MMSE scores were treated as a continuous variable, it was standardized by z-score, and a Per-SD was 1.00.

a An unadjusted model.

b An adjusted for age, gender, and residence.

c Further adjusted for education level, marital status, smoking status, alcohol drinking, and exercise.

d Further adjusted for BMI, physical impairment, dementia history, multimorbidity, WC, SBP, DBP, TG, HDL-C, LDL-C, FPG, HbA1c, and uric acid.

*P < 0.001; **P < 0.01.

### MMSE sub-domains and mortality

The ability to predict mortality not only exists in overall cognitive function but in several MMSE domains. Table 3 shows the associations between MMSE sub-domains and all-cause and CVD mortality. In the fully adjusted model, time orientation (HR, 1.16; 95% CI: 1.04–1.29; *P* = 0.009) and calculation and attention (HR, 1.31; 95% CI: 1.11–1.54; *P* = 0.001) were significantly associated with all-cause mortality. The increased risk of CVD mortality was also associated with orientation to time (HR, 1.26, 95% CI: 1.08–1.47; *P* < 0.004), orientation to place (HR, 1.14, 95% CI: 1.01–1.28; *P* < 0.032), and calculation and attention (HR, 1.51, 95% CI: 1.17–1.95; *P* < 0.002). Other MMSE sub-domains were not associated with all-cause and CVD mortality.

**Table 3:** Hazard ratio of all-cause and CVD mortality among MMSE sub-domains.

**Item**	**Model 1^a^**	**Model 2^b^**	**Model 3^c^**	**Model 4**
	**HR (95%CIs)**	**HR (95%CIs)**	**HR (95%CIs)**	**HR (95%CIs)**
All-cause mortality
MMSE domains^†^				
Orientation to time	1.53 (1.39–1.69)*	1.21 (1.09–1.34)*	1.15 (1.04–1.29)**	1.16 (1.04–1.29)**
Orientation to place	1.23 (1.14–1.33)*	1.08 (0.99–1.17)	1.04 (0.95–1.14)	1.04 (0.95–1.14)
Immediate recall	1.11 (0.98–1.25)	1.11 (0.98–1.25)	1.07 (0.95–1.20)	1.06 (0.94–1.19)
Delayed recall	1.40 (1.29–1.52)*	1.09 (1.09–1.11)	1.08 (0.98–1.18)	1.09 (0.99–1.20)
Language and others	1.36 (1.23–1.50)*	1.14 (1.02–1.26)***	1.08 (0.96–1.20)	1.08 (0.97–1.21)
Calculation and attention	1.26 (1.34–1.82)*	1.33 (1.13–1.56) *	1.31 (1.12–1.55) **	1.31 (1.11–1.54) **
Cardiovascular disease mortality
MMSE domains^†^				
Orientation to time	1.78 (1.56–2.04)*	1.32 (1.13–1.52)*	1.28 (1.10–1.49)**	1.26 (1.08–1.47)**
Orientation to place	1.38 (1.26–1.52)*	1.18 (1.06–1.32)***	1.16 (1.03–1.30)***	1.14 (1.01–1.28)***
Immediate recall	1.17 (0.98–1.40)	1.16 (0.98–1.38)	1.12 (0.94–1.33)	1.10 (0.92–1.31)
Delayed recall	1.56 (1.40–1.74)*	1.18 (0.98–1.40)***	1.15 (1.00–1.31)***	1.15 (1.00–1.31)
Language and others	1.53 (1.33–1.75)*	1.22 (1.05–1.42)***	1.16 (0.99–1.36)	1.14 (0.97–1.34)
Calculation and attention	1.87 (1.47–2.39)*	1.52 (1.19–1.95)**	1.52 (1.18–1.96)**	1.51 (1.17–1.95)**

CIs, confidence intervals; CVD, cardiovascular disease.^†^Score was converted to Z standard score, which represented higher score to worse performance.

^a^An unadjusted model.

^b^An adjusted for age, gender, and residence.

^c^Further adjusted for education level, marital status, smoking status, alcohol drinking, and exercise.

^d^Further adjusted for BMI, physical impairment, dementia history, multimorbidity, WC, SBP, DBP, TG, HDL-C, LDL-C, FPG, HbA1c, and uric acid.

*P < 0.001; **P < 0.01,***P < 0.05.

### Subgroup and sensitivity analysis

**Figure 4:**
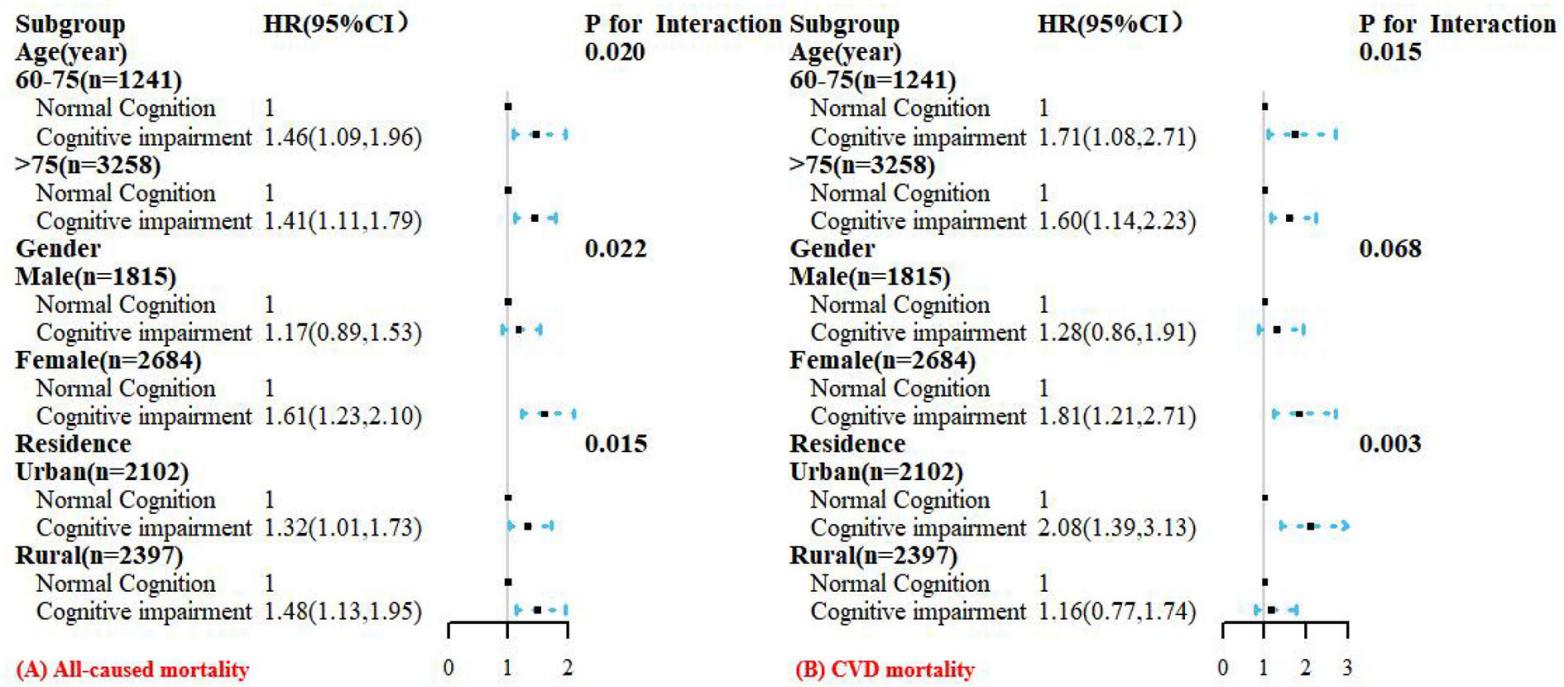
Hazard ratios (HRs) for the associations between cognitive impairment with all-cause **(A)** and CVD **(B)** mortality according to age, gender, and residence subgroups. All models were adjusted for age (not in age subgroup), gender (not in gender subgroup), residence (not in residence subgroup), education, marital status, smoking status, alcohol drinking, exercise; BMI, physical impairment, dementia history, multimorbidity, WC, SBP, DBP, TG, HDL-C, LDL-C, FPG, HbA1c, and uric acid.

Consistent results were observed in each subgroup analysis (Figure 4). In the age subgroup, a stronger positive association was found in younger participants (all interactions *P* < 0.05). Similar results were displayed in the gender subgroups, and female participants with CI had a higher risk of all-cause (interaction *P* < 0.05) and CVD mortality than male participants. In addition, analysis stratified by residence showed that participants with CI in rural regions had a higher risk of all-cause mortality than urban participants, but a lower risk of CVD mortality than urban participants (all interactions *P* < 0.05). We also conducted stratified analysis by baseline chronic diseases and found that CI was significantly positively associated with all-cause and CVD mortality among participants with self-reported hypertension or diabetes (all interactions *P* < 0.05, Supplementary Tables S4, S5).

Regarding sensitivity analyses, the trend of overall effect was unchanged essentially when excluding 95 participants who died within 2 years of follow-up (Table 4). In addition, similar findings were observed when considering participants with a history of CVD, but the risk of all-cause and CVD mortality was further increased for participants with CVD and CI.

**Table 4:** Sensitivity analysis of the association between cognitive function and all-cause and CVD mortality.

**MMSE groups**	**All-cause mortality**			**Cardiovascular disease mortality**		
	**Model 1^a^**	**Model 2^b^**	**Model 3^c^**	**Model 1^a^**	**Model 2^b^**	**Model 3^c^**
	**HR (95%CIs)**	**HR (95%CIs)**	**HR (95%CIs)**	**HR (95%CIs)**	**HR (95%CIs)**	**HR (95%CIs)**
Participants with follow-up time over 2 years, *n*/total = 572/4404						
Normal cognition Cognitive impairment	1 1.86 (1.54, 2.25) *	1 1.31 (1.07, 1.61)**	1 1.30 (1.06, 1.59) **	1 2.20 (1.67, 2.89)*	1 1.36 (1.01, 1.83) **	1 1.36 (1.01, 1.84)**
Per-SD increase	0.72 (0.64, 0.80)*	0.78 (0.69, 0.89)*	0.85 (0.77, 0.94)**	0.58 (0.50, 0.67) *	0.63 (0.53, 0.75) *	0.75 (0.65, 0.86) *
Participants with cardiovascular disease, n/total=659/3182						
Normal cognition Cognitive impairment	1 1.99 (1.64, 2.42) *	1 1.45 (1.17, 1.78)**	1 1.45 (1.17, 1.81) **	1 2.23 (1.69, 2.94)*	1 1.47 (1.09, 1.99) **	1 1.51 (1.11, 2.07)**
Per-SD increase	0.69 (0.64, 0.73)*	0.78 (0.71, 0.86)*	0.84 (0.75, 0.93)**	0.61 (0.55, 0.68) *	0.70 (0.62, 0.80) *	0.74 (0.63, 0.86) *

MMSE, Mini-Mental State Examination; CI, confidence interval; SD: standard deviation; CVD, cardiovascular disease. When MMSE scores were treated as a continuous variable, it was standardized by z-score, and a Per-SD was 1.

a An unadjusted model.

b An adjusted for age, gender, and residence.

c Further adjusted for education, marital status, smoking status, alcohol drinking, exercise, BMI, physical impairment, dementia history, multimorbidity, WC, SBP, DBP, TG, HDL-C, LDL-C, FPG, HbA1c, and uric acid.

* P < 0.001; **P < 0.05.

## Discussion

In this prospective study of 4,499 Chinese older adults, we evaluated the relationship between cognitive function and the risk of all-cause and CVD mortality, and the results showed that the risk of all-cause mortality and CVD mortality was significantly increased in the CI group compared with cognitively normal participants. The association remained significant even after several sensitivity analyses and adjustments for some potential explanatory factors, including demographic factors, BMI, lifestyle, multimorbidity, and biochemical indexes. Furthermore, place orientation, time orientation, and calculation and attention in MMSE were significantly associated with mortality. The findings confirmed that CI is a predictor of all-cause and CVD mortality in the Chinese population, independently of other common risk factors such as hypertension, diabetes, and BMI.

Epidemiological studies in developed countries have extensively reported a strong association between CI evaluated by MMSE and increased risk of all-cause mortality in older adults (5–12). To the best of our knowledge, there are also a few independent studies that have investigated the association in China (13, 15, 16, 19, 20, 28). However, education-adjusted MMSE cutoff scores were not widely used to define CI in these studies, education level is the largest factor influencing the bias of MMSE scores, screening subjects with different levels of education using the same cutoff value will affect the validity of the measure (25, 26), and the results may not be directly comparable (15, 16, 20). In developing countries like China, illiteracy is still widely prevalent, particularly among the elderly population; therefore, using education-adjusted cutoffs is important to decrease the chance of false positives (27). Two studies in China reported that CI detected by education-adjusted MMSE cutoff scores was associated with the increases in all-cause mortality by 53% in participants aged ≥80 years and 80% in individuals aged 65 or over, respectively (13, 28). Our results are consistent with these studies reported (13, 28), but the risk of all-cause mortality is relatively low. Two main reasons exist: One is the inclusion of as many covariates as possible, which is the most comprehensive and sufficient factor compared to existing studies (10–12). Second, the reference group chosen was different. Although cognitively normal participants are used as the reference group, the number of people grouped under different delineation criteria will affect the value of the hazard ratios (13, 20, 28).

Many population-based studies have reported the association between CI and subsequent increased risk of CVD mortality among elderly individuals (8–12), though the relationship is complex and the potential mechanisms remain unclear. Our findings that CI as a proxy measure can predict CVD mortality are consistent with some previous studies in Western populations (10, 11, 25). Three European studies found a 1.35- to 1.71-fold higher risk of CVD mortality for individuals with CI compared with cognitively normal peers (8–10), and the magnitudes differ due to several reasons, including different populations (8–10), differences in the measurement tools used to assess cognitive function status (8, 11), non-uniform definitions of CI (8–10), and short follow-up time (8–11). However, some studies found the association only held in minimally adjusted models (11, 12), while our study found that the association between CI and CVD mortality was consistent in both unadjusted and fully adjusted models. Furthermore, we investigated this association in participants with cardiovascular disease separately in sensitivity analysis and found that CI further enhanced the risk of CVD mortality. This analysis showed that vascular events and CI share similar risk factors and interact to increase the risk of mortality (21, 29, 30). Because the relationship between vascular events and CI seems to be closely intertwined, it is hard to disentangle them and confirm a causal direction within the link. This study provided further evidence to study the implication of the interaction between CI and vascular diseases.

The association between the sub-domains of MMSE and mortality is not established. One study in Japanese found an association between mortality with MMSE sub-domains for time orientation, recall of words, naming objects, place orientation, and listening and obeying (22), and another study in Japanese community-dwelling older adults reported that place orientation, calculation, and delayed recall among the MMSE sub-domains were significantly associated with all-cause mortality (23). Similarly, the study in Koreans indicated that time orientation and calculation of MMSE sub-domains were associated with increased all-cause mortality (24). In our study, we found that MMSE sub-domains (apart from immediate recall) were significantly associated with all-cause and CVD mortality, but after adjusting for confounders, only orientation and calculation and attention were still independently associated with mortality. It has been shown that time orientation and calculation and attention were effective domains for predicting total MMSE score, and these domains have a significant correlation with daily functioning (20, 31, 32). Time orientation is the earliest lost domain in dementia and the most useful domain to distinguish between normal and demented individuals when assessing age-related decline (32). The calculation and attention domain is considered the most valuable and particularly useful in older adults, but the skills required to properly perform this domain are prone to degradation during normal aging. These domains require both episodic and semantic memory, and deficits in these domains were seen as hallmarks of early AD (24, 30). However, a study in urban community-dwelling Chinese older adults found an association between MMSE sub-domains and all-cause mortality in the unadjusted model, but not when additionally adjusting for age and gender, possibly due to differences in the criteria used to define impaired MMSE sub-domains (21–24), age group, and potential confounders (20, 31, 32). MMSE sub-domains were not independent of each other, and any particular test requires a range of abilities, a single-item test cannot give a measure of overall cognitive ability. Hence, further research is needed to confirm whether some domains are more strongly related to mortality than others.

Previous studies have demonstrated that age and gender were significant modifiers of the association between CI and mortality (13, 14, 33–35). When the association was stratified by age in our study, we found positive associations in different age groups, and the association seemed stronger in younger participants compared to the older ones. This may be caused by “survivor bias” (17). As an older group (age>75) who may already have CI, this group has gradually adapted to current cognitive function status compared with the younger older adults who may more sensitive to the association with reduced MMSE scores. Similarly, there are gender differences between CI and all-cause and CVD mortality. It is consistent with Kirsten's report (35), which may be caused by the particularity of women. Women performed worse than men in terms of lifelong emotional disorders or subnormal cognitive functioning. However, participants with CI in rural regions had a higher risk of all-cause mortality than urban participants, but the risk of CVD mortality was lower than urban participants. It may be related to the higher economic level and medical care in urban regions, which reduces the risk of all-cause mortality. In contrast, differences in diet structure, daily exercise, chronic diseases, and air pollution contribute to a higher risk of CVD mortality in urban regions. It is necessary to further explore the patterns of death and possible influencing factors for individuals with CI. We also found that the risk of all-cause mortality and CVD mortality was higher in the diabetes subgroup than in the non-diabetes subgroup. These findings suggested that controlling these common risk factors may have important implications in reducing mortality risk.

It is unclear why individuals with CI are more likely to live shorter. However, it is generally believed that the possible potential mechanisms are as follows: The first explanation is that CI may reflect “terminal decline,” which is a marker of their own increased potential diseases and decline of health status (36). As the skills required for medical and health knowledge acquisition and integrated health information are directly related to cognitive function, individuals with CI may be difficult to recognize the symptoms of the disease, receive diagnosis and treatment in the early stage of life, and follow the advice of doctors, causing disease deterioration and further reducing life span (37). The second explanation is system integrity, which postulates that good cognitive function may mark a better constitution (38). A better cognitive function might be a “well-wired” physiological trait that enables stronger responses to common environmental stress. The third explanation is the frailty hypothesis that CI reflects the collapse of multiple physiological systems due to the accumulation of aging-related chronic diseases, leading to organ failure and failure of homeostatic systems (39), which were thought to be proximal specific factors that occur later in life and were key factors leading to cognitive decline and subsequent death (40). Finally, it is worth mentioning the potential role of genes (Apolipoprotein E) in the association between CI and mortality. The ApoE allele variation is implicated in many common illnesses including cognitive decline measured by MMSE and AD (41), and there is a synergistic effect between ApoE4 carrier status and cognitive status in relation to mortality (42). We explored whether demographic and lifestyle factors may influence the association between CI and mortality. In particular, we also evaluated the influence of hypertension and diabetes, since we were unable to obtain information on socioeconomic status and depressive symptoms that may confound the association. However, these factors are potentially modifiable, and understanding their contribution to the association between cognition and mortality is of clinical importance.

Our study has some valuable strengths. First, the data that we analyzed were prospectively collected, including individuals from urban and rural regions. Our study included a broader age range of older adults (60–95 years old), which is easier to generalize to the general older adults in China. Second, a variety of potential explanatory factors were carefully considered, and different adjustment strategies were presented to ensure the authenticity of the results. Considering multimorbidity is common in older adults and may add a short-term risk of death rather than cognition accounting for the spurious relationship, we examined it using sensitivity analyses. Third, these urban and rural community-dwelling older adults represent the cognitive function level in the modern economic and cultural level in Beijing, and 58.3% of the older adults have received primary school or above.

There are several limitations. First, cognitive function was only assessed at baseline, and the changes in cognitive function may be as important as baseline cognitive function because these two measures could lead to different outcomes (15, 43). However, our follow-up did not include newly diagnosed CI, and we will further explore whether the longitudinal changes in cognitive function are related to the increased risk of mortality in the future. Second, there might be residual confounding bias that inevitably influences the outcomes, though lots of covariates were considered, some potentially relevant factors were not taken into accounts, such as depressive symptoms which can influence the CI and death (9, 44). However, we comprehensively adjusted for all possible explanatory factors and the results were still robust. Third, given the possible high correlation of MMSE sub-domains, the application and promotion of outcomes need further verification in clinical studies and population analysis. Finally, the participants were not representative of older adults in China, and the results should be further confirmed in subjects from other geographic and ethnic groups of China.

CI assessed by MMSE is a marker of mortality in Chinese older adults, but further research is needed to identify the mechanisms underlying the association. It could not evaluate whether changes in cognitive trajectory were associated with an elevated risk of mortality because cognitive impairment in this study was based on a single measure. Repeated measurements of MMSE during long-term follow-up are more valuable for analyzing cognitive trajectory transition and mortality. Furthermore, exploring the MMSE sub-item sensitive to mortality is meaningful to confirm whether some domains are more strongly related to mortality than others.

## Conclusion

This study revealed that CI is a marker of all-cause and CVD mortality risk in Chinese older adults, particularly in women and younger older adults. Orientation and calculation and attention were strongly associated with mortality, but further research is warranted. Given the suggestion that CI is modifiable, strengthening the treatment and management of the condition of patients with diabetes and enhancing early screening and intervention for impaired cognition in older adults are important to reduce the risk of mortality and promote the formulation of longevity strategies.

## Data availability statement

The raw data supporting the conclusions of this article will be made available by the authors, without undue reservation.

## Ethics statement

The studies involving human participants were reviewed and approved by the Ethics Committee of Chinese PLA General Hospital. The patients/participants provided their written informed consent to participate in this study.

## Author contributions

ZL, ML, YH, and YoW contributed to the conception and design of the study. XG, SL, YaW, DW, MY, and SC managed the data and provided help in the data analysis. ZL, ML, and SW performed the statistical analysis and wrote the first draft of the manuscript. RL, HL, XL, RJ, XZ, and JG made corrections and suggestions on the defects of the first draft. All authors critically reviewed draft versions, provided important intellectual content during revisions, and accepted accountability for the overall work.

## Funding

The study was supported by the Program of the National Natural Science Foundation of China (Serial No.: 82173589,82173590) and the Special Grant for the Prevention and Control of Infectious Diseases (2018ZX10713003).

## Conflict of interest

The authors declare that the research was conducted in the absence of any commercial or financial relationships that could be construed as a potential conflict of interest.

## Publisher's note

All claims expressed in this article are solely those of the authors and do not necessarily represent those of their affiliated organizations, or those of the publisher, the editors and the reviewers. Any product that may be evaluated in this article, or claim that may be made by its manufacturer, is not guaranteed or endorsed by the publisher.
