# Supplementary material for: Cognitive impairment assessed by Mini-Mental State Examination predicts all-cause and CVD mortality in Chinese older adults: A 10-year follow-up study
Source: Front Public Health. 2022 Nov 28;10:908120. doi: 10.3389/fpubh.2022.908120 (PMC9744251; doi:10.3389/fpubh.2022.908120)
Supplement: Supplementary file 1 [file Table_1.docx]

**Supplementary materials**

### Figure S1. Flowchart of the inclusion and exclusion of participants.

### Table S1. The Chinese version of the Mini-Mental State Exam (MMSE)

Table S2. Survival Status based on different cognitive function status

Table S3. Summary of clinical measurements collected in the baseline of the BECHCS

Table S4. Hazard ratios for the association between Cognitive impairment with all-cause and CVD mortality by hypertension or not

Table S5. Hazard ratios for the association between Cognitive impairment with all-cause and CVD mortality by diabetes or not

Table S6. Hazard ration for the association between Cognitive impairment and Non-CVD mortality


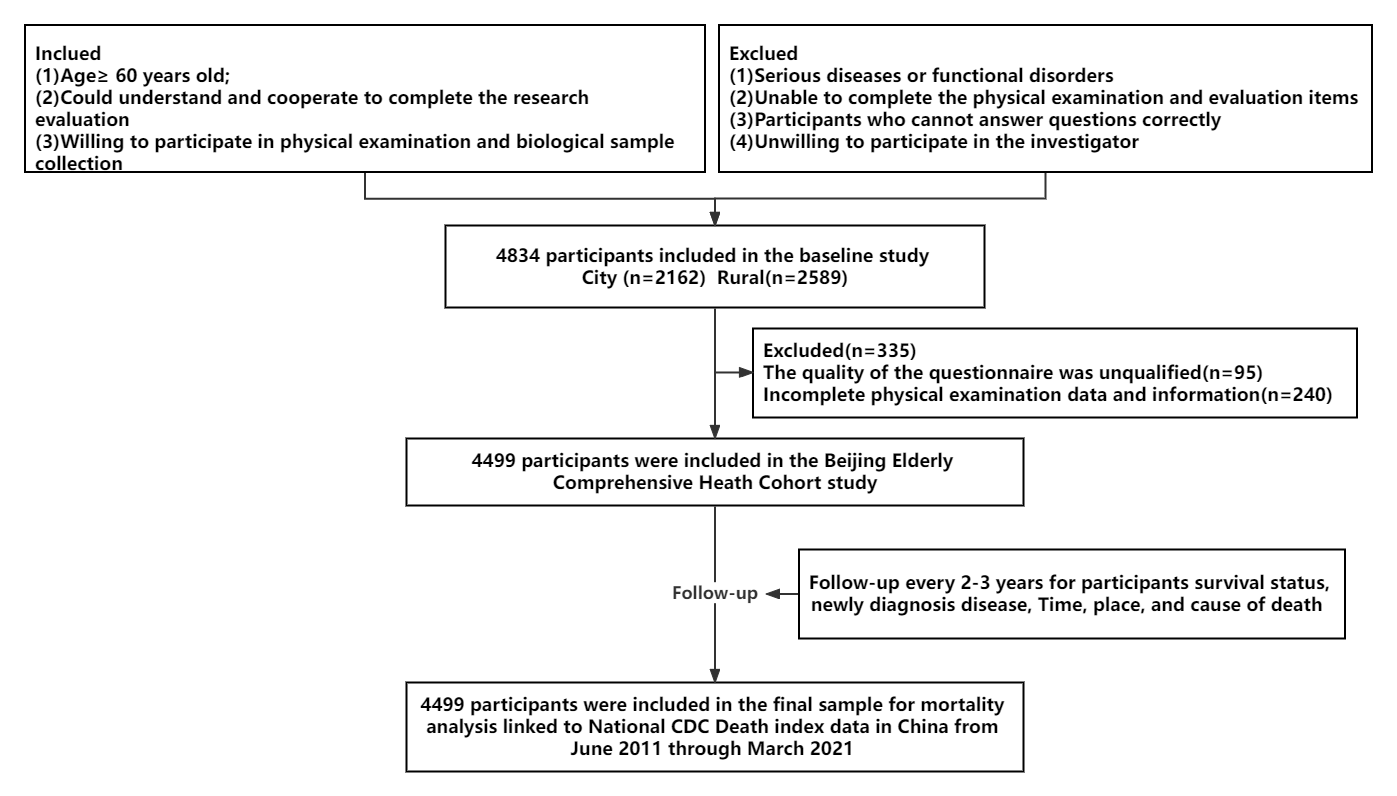


### Figure S1. Flowchart of the inclusion and exclusion of participants.

| **Table S1. The Chinese Version of the Mini-Mental State Exam (MMSE)** | | |
| --- | --- | --- |
| Item | MMSE | Score (Total =30) |
| Time Orientation | What day is today? | 1 |
|  | What is the animal year of this year? | 1 |
|  | What is the date (day and month) right now? | 2 |
|  | What is the season right now? | 1 |
| Place Orientation | Where are we now? | 1 |
|  | Which street are we stay now? | 1 |
|  | Which floor are we stay now? | 1 |
|  | What is the name of this city and country? | 2 |
| Calculation and attention | Would you please calculate the number 100 minus 7? Then reduce seven in a row, and the number you get is reduced in a row (five times in total) | 5 |
|  | Please count down from 10 to 1 | 1 |
| Immediate recall | keys, vase, and ruler. Please repeat these three objects. | 3 |
| Delayed recall | Name the three objects learned earlier (keys, vase, and ruler). | 3 |
| Language | Please read this sentence on the paper(close your eyes)and do it as it means | 1 |
|  | Please name the two substances presented by the observer（watch，pen） | 2 |
| Writing and executive | Please write down your name. | 1 |
|  | The participant is asked to draw a figure of overlapping pentagons. | 1 |
|  | The participant is asked to follow the interviewer’s instruction: “Take the paper using your right hand, fold it in the middle using both hands, and place the paper on the legs.” | 3 |

| **Table S2.Summary of clinical measurements collected in the baseline of the BECHCS** | | | |
| --- | --- | --- | --- |
| Variables | Number of measurements | Equipment  (or biochemical analyzer) | From which City |
| Standing height  Weight  Waist and Hip circumference  Resting blood pressure  Complete blood count ^a^  Comprehensive metabolic biochemical ^b^  Lipid profile ^c^  Urinalysis ^d^ | ONE  ONE  TWO (Each)  TWO  ONE  ONE  ONE  ONE | Manufactured instrument  Manufactured instrument  Tape measurement  Omron HEM-7200  HITACHI 7080  Roche Cobas 8000  HITACHI 7080  Changchun Dirui H-800 | China  China  China  China  Japan  Switzerland  Japan  China |
| a. White blood cell counts and red blood cell counts, neutrophil count, platelets (PLT) count and white blood cell count.  b. Electrolytes, fasting plasma glucose (FPG), glycosylated hemoglobin A1c (HbA1c), serum uric acid, blood urea nitrogen, creatinine, uric acid (UA) and serum total protein (TP),  c. Total cholesterol (TC), Triglyceride (TG), Apolipoproteins, Low-density lipoprotein cholesterol (LDL-C) and High-density lipoprotein cholesterol (HDL-C).  d. pH value (pH), urinary microalbumin (MALB), Urine specific gravity (USG). | | | |

| **Table S4. Hazard ratios for the association between Cognitive impairment with all-cause and CVD mortality by hypertension or not** | | | | | |
| --- | --- | --- | --- | --- | --- |
|  | **Non-hypertension** | | **Hypertension** | | **P for interaction** |
|  | **Normal cognition** | **Cognitive impairment** | **Normal cognition** | **Cognitive impairment** |  |
| Participants, No. | 1260 | 271 | 2320 | 648 |  |
| **All-cause mortality** | | | | | |
| Death, No. | 152 | 53 | 331 | 131 |  |
| Model 1^a^ | 1(ref) | 1.19(0.85,1.67) | 1(ref) | 1.44(1.16,1.80) ** | <0.001 |
| Model 2^b^ | 1(ref) | 1.17(0.83,1.66) | 1(ref) | 1.36(1.08,1.70) ** | 0.001 |
| Model 3^c^ | 1(ref) | 1.15(0.81,1.64) | 1(ref) | 1.38(1.10,1.73) ** | 0.010 |
| **CVD mortality** | | | | | |
| Death, No. | 47 | 26 | 154 | 65 |  |
| Model 1^a^ | 1(ref) | 1.70(1.01,2.88) *** | 1(ref) | 1.40(1.02,1.93) *** | <0.001 |
| Model 2^b^ | 1(ref) | 1.86(1.09,3.17) *** | 1(ref) | 1.32(0.98,1.78) | 0.001 |
| Model 3^c^ | 1(ref) | 1.76(1.01,3.08) *** | 1(ref) | 1.37(0.99,1.90) | 0.026 |
| Note: CVD, cardiovascular disease; HR, hazard ratio.  a Adjusted for age, gender and residence  b Further adjusted for education level, marital status, smoking status, alcohol drinking, exercise;  c Further adjusted for BMI, physical impairment, dementia history, multimorbidity(without hypertension**)**,WC, SBP, DBP, TG，HDL-C，LDL-C, Uric acid.  * P < 0.001; **P < 0.01；***P < 0.05 | | | | | |

| **Table S3. Status of survival based on different cognitive function status (n=4499,%)** | | | | | | | | | | | |
| --- | --- | --- | --- | --- | --- | --- | --- | --- | --- | --- | --- |
| Status of survival | Female | | |  | Male | | | Total  (n=4499) | P-value | | |
|  | Age ≥75 | Age<75 | Total  (n=1787) |  | Age ≥75 | Age<75 | Total  (n=1815) |  | P_a_ | P_b_ | P_c_ |
| **Death** | | | | | | | | | | | |
| Normal cognition  CI | 91(60.3)  60(37.9) | 120(73.6)  43(26.4) | 211(67.2)  103(32.8) |  | 147(70.0)  63(30.0) | 125(87.4)  18(12.6) | 272(77.1)  81(22.9) | 483(72.4)  184(27.6) | <0.001 | <0.001 | <0.001 |
| Alive | | | | | | | | | | | |
| Normal cognition  CI | 315(63.8)  179(36.2) | 1541(82.1）  335(17.9) | 1856(78.3)  514(21.7) |  | 297(76.9)  89(23.1) | 944(87.7)  132(12.3) | 1241(84.9)  221(15.1) | 3097(80.8)  735(19.2) | <0.001 | <0.001 | <0.001 |
| Note :CI，Cognitive impairment.P_a_ is the comparison between male and female; P_b_ is the comparison of all ages in the female group; P_C_ is the comparison of all ages in the male group Percentage is the proportion of rows | | | | | | | | | | | |

| **Table S5.Hazard ratios for the association between Cognitive impairment with all-cause and CVD mortality by diabetes or not** | | | | | |
| --- | --- | --- | --- | --- | --- |
|  | **Non-diabetes** | | **Diabetes** | | **P for interaction** |
|  | **Normal cognition** | **Cognitive impairment** | **Normal cognition** | **Cognitive impairment** |  |
| Participants, No. | 2789 | 721 | 791 | 198 |  |
| **All-cause mortality** | | | | | |
| Death, No. | 342 | 124 | 141 | 60 |  |
| Model 1^a^ | 1(ref) | 1.22(0.98,1.53) | 1(ref) | 1.87(1.35,2.59) * | <0.001 |
| Model 2^b^ | 1(ref) | 1.16(0.93,1.46) | 1(ref) | 1.89(1.37,2.62) * | <0.001 |
| Model 3^c^ | 1(ref) | 1.16(0.92,1.45) | 1(ref) | 1.85(1.32,2.59) * | <0.001 |
| **CVD mortality** | | | | | |
| Death, No. | 140 | 60 | 61 | 31 |  |
| Model 1^a^ | 1(ref) | 1.23(0.88,1.72) | 1(ref) | 2.33(1.46,3.71) * | <0.001 |
| Model 2^b^ | 1(ref) | 1.20(0.85,1.68) | 1(ref) | 2.45(1.54,3.91) * | <0.001 |
| Model 3^c^ | 1(ref) | 1.20(0.86,1.69) | 1(ref) | 2.42(1.48,3.97) * | <0.001 |
| Note: CVD, cardiovascular disease; HR, hazard ratio.  a Adjusted for age, gender and residence  b Further adjusted for education level, marital status, smoking status, alcohol drinking, exercise;  c Further adjusted for BMI, physical impairment, dementia history, multimorbidity(without diabetes**)**,WC, TG，HDL-C，LDL-C, ,FPG,HbA1c,Uric acid.  * P < 0.001; **P < 0.01；***P < 0.05 | | | | | |

| **Table S6. Hazard ration for the association between Cognitive impairment and Non-CVD mortality** | | | |
| --- | --- | --- | --- |
|  | Model 1^a^ | Model 2^b^ | Model 3^c^ |
|  | HR (95%CIs) | HR (95%CIs) | HR (95%CIs) |
| **Cancer mortality** | | | |
| **Cut-point for education**  Normal cognition  Cognitive impairment | 1(Reference)  1.36(0.97-1.88) | 1(Reference)  1.08(0.76-1.53) | 1(Reference)  1.08(0.59-1.26) |
| Per-SD increase | 1.03(0.88-1.22) | 1.07(0.89-1.28) | 1.07(0.89-1.28) |
| **Respiratory mortality** | | | |
| **Cut-point for education**  Normal cognition  Cognitive impairment | 1(Reference)  1.04(0.52-2.08) | 1(Reference)  1.03(0.51-2.09) | 1(Reference)  1.03(0.51-2.12) |
| Per-SD increase | 0.73(0.57-0.95) * | 0.85(0.64-1.14) | 0.85(0.64-1.14) |
| Note: CIs indicates confidence intervals; Hazard ratio (95% CI) was calculated from Cox models.  a Adjusted for age, gender and residence  b Further adjusted for education level, marital status, smoking status, alcohol drinking, exercise;  c Further adjusted for BMI, physical impairment, dementia history, multimorbidity (without diabetes**)**,WC, SBP, DBP, TC, TG，HDL-C，LDL-C, ,FPG,HbA1c,Uric acid.  *P<0.05； | | | |
